# A Role for Non-Antimicrobial Actions of Tetracyclines in Combating Oxidative Stress in Periodontal and Metabolic Diseases: A Literature Review

**DOI:** 10.2174/1874210600802010005

**Published:** 2008-01-22

**Authors:** M Soory

**Affiliations:** Periodontology, King’s College London Dental Institute at G KT Hospitals, King’s College Dental Hospital, Denmark Hill, London SE 5 9RW, UK

**Keywords:** Inflammatory diseases, oxidative stress, antioxidants, tetracyclines

## Abstract

This review addresses the role of adjunctive tetracycline therapy in the management of periodontal diseases and its efficacy in reducing inflammatory burden, oxidative stress and its sequelae in patients with coexisting features of metabolic syndrome. Removal of the dimethylamine group at C4 of the tetracycline molecule reduces its antibiotic properties, enhancing its non-antimicrobial actions; this strategy has aided the development of several chemically modified tetracyclines such as minocycline and doxycycline, by altering different regions of the molecule for focused action on biological targets. Tetracyclines are effective in reducing inflammation by inhibiting matrix metalloproteinases, preventing excessive angiogenesis, inhibiting apoptosis and stimulating bone formation. There are important applications for tetracyclines in the management of diabetic, dyslipidaemic periodontal patients who smoke. The diverse mechanisms of action of tetracyclines in overcoming oxidative stress and enhancing matrix synthesis are discussed in this review.

## INTRODUCTION

There is increasing awareness of the role of oxidative stress in causing significant damage to hard and soft tissues. The relevance of oxidative stress induced tissue damage as a common nucleus of disease progression in periodontal and metabolic diseases is highlighted in this review. A therapeutic role for the non-antimicrobial actions of tetracyclines is discussed in this context.

Periodontal disease has a microbial aetiology and an inflammatory pathogenesis, characterised by destruction of tooth-supporting soft and hard tissues of the alveolus resulting in periodontal pockets and roots denuded of periodontal ligament, cementum and alveolar bone. Periodontal patients have been reported to have a higher prevalence of systemic diseases than a general dental population and are more likely to be on medication for multiple ailments [1] Modulation of systemic diseases such as diabetes, also associated with a substantial inflammatory burden could affect the progression of periodontal disease [2-4]. Prolonged hyperglycaemic spells are likely to be a contributory factor, considering the frequency and severity of periodontal disease in those with poor glycaemic control [5]. Specific periodontal pathogens and their products associated with host virulence can contribute to a significant systemic inflammatory burden [6-8]. Smokers are more at risk of developing severe periodontal disease than non-smokers [9, 10]. It is relevant that uncontrolled diabetes mellitus is associated with more severe destructive periodontal disease, worse in smokers [11].

Effective management of severe periodontal disease can reduce the inflammatory burden in this population of patients. This has been demonstrated with regard to reduced insulin resistance in diabetic patients following effective periodontal management [12]. Raised levels of C- reactive proteins (CRP) amongst periodontal patients and those with acute myocardial infarction has been reported, independent of other risk factors [13]. Three months after intensive periodontal treatment in patients with a proven history of cardiovascular disease there was significant reduction in levels of CRP and oxidised low density lipoprotein [14]; associated host responses contribute to the manifestations of clinical parameters of periodontal disease [15]. The link between cardiovascular and periodontal diseases is well documented while a causal relationship is more difficult to establish. A generalised distribution of severe periodontal disease is likely to contribute to significant inflammatory loading in subjects with systemic chronic inflammatory diseases. There is potential for adjunctive therapeutic management of periodontal diseases with the tetracycline group of drugs and other host modulatory agents used to arrest an over exuberant inflammatory response [16]. They have beneficial effects on systemic diseases which are also driven by oxidative stress; in view of their antioxidant, anti-inflammatory and proanabo-lic effects [17] in addition to their anti-microbial actions.

These non-antibiotic properties of tetracyclines also include immunomodulatory, angiogenic and anti-apoptotic effects. There are 3 main groups of tetracyclines consisting of the natural product, semi-synthetic compounds and the chemically modified tetracyclines [18]. The tetracyclines have a chemical structure consisting of a tetracyclic naphthacene carboxamide ring system. The dimethylamine group at carbon 4 (C4) in ring A confers antibiotic properties on the drug and its removal has the advantage of reducing these effects and also enhancing its non-antibiotic actions [18]. This is used to advantage in several other applications of such chemically modified tetracyclines. Biological targets of action of tetracyclines can be enhanced by modifying ring D through carbon locations C7-C9. This is the basis of efficacy of the semisynthetic compounds minocycline and doxycycline. Their anti-inflammatory effects are initiated by several mechanisms. They inhibit matrix metalloproteinases (MMPs). MMPs are zinc-dependent endopeptidases which play an important role in remodelling of connective tissue matrices in wound healing and rheumatoid arthritis.

## APPLICATIONS FOR ADJUNCTIVE USE OF TETRACYCLINES AS ANTIMICROBIALS IN THE MANAGEMENT OF PERIODONTAL DISEASES

The initiation and progression of periodontal diseases are dependent on bacterial plaque. Treatment strategy is based on controlling pathogenic potential to sub-threshold levels for disease production. This is achieved predominantly by carrying out thorough periodontal root surface debridement. Adjunctive antimicrobials have been used over the past 20 years in the management of periodontal diseases. They are particularly useful in the management of aggressive forms that present with severe disease for age in susceptible individuals [19, 20]. Other indications are recalcitrant sites common in smokers [21, 22] which may not have responded to conventional root surface debridement and also in the management of acute periodontal conditions. It is often a matter of clinical judgement with regard to choosing the best treatment option for a particular mode of disease presentation.

A systematic review comprising a meta-analysis of 19 studies showed that combining root surface debridement with sustained release antimicrobials was significantly more effective in resolving periodontal disease than root surface debridement alone [23]. Minocycline gel, microencapsulated minocycline, chlorhexidine chip and doxycycline gel were sustained delivery systems used for placement in the periodontal pocket. It was also shown that the effect of antimicrobial agents alone was comparable to that of root surface debridement alone. It must be emphasised that judicious use of antimicrobials is essential. They are best reserved for at risk young populations, those compromised with systemic diseases which accelerate periodontal disease progression, those who have not responded to conventional measures and those presenting with acute periodontal conditions; for adjunctive use and not as a substitute for root surface debridement, compatible with current standards of care.

Other workers reinforce this view. Periodontal root surface debridement with adjunctive application of minocycline microspheres in periodontal pockets has been shown to consistently reduce parameters of site based periodontal disease compared with root surface debridement alone in a multicentre randomised trial of 499 patients [24]. Similar results have been reported in a split mouth study of 15 patients over 18 weeks with significant improvement in periodontal paramenters and IL-1β content, when root surface debridement was combined with adjunctive application of minocycline [25]. Minocycline hydrochloride microspheres have also been shown to be effective against peri-implantitis [26]; there was a significant reduction in the periodontal pathogenic load, particularly of *Aggregatibacter actinomycetemcomitans* over a period of one year. In view of microbial resistance caused by excessive use of antimicrobials one would need to consider the efficacy of thorough mechanical root surface debridement in reducing microbial loading to sub-threshold levels for disease production; and utilise the non-antimicrobial formulations of tetracyclines as adjuncts for their actions detailed in the sections that follow.

Long-term usage of antimicrobial formulations of tetracyclines in current and past users could contribute to hepatotoxicity. Doxycycline is potentially less hepatotoxic than tetracycline and could be a safer substitute where appropriate [27]. Their applications as adjuncts to periodontal management are relatively short term and less likely to induce toxicity or microbial resistance, in comparison with those for chronic conditions such as acne and rosacea. Stringent surveillance and selective usage limited to cases that require this intervention must be practised in order to minimise the development of microbial resistance. An *In vitro* study was done to investigate the effects of minocycline and doxycycline on human gingival fibroblasts, epithelial cells and periodontal ligament fibroblasts with regard to cell survival and protein expression genes [28]. The concentrations used were greater than minimum inhibitory concentrations for 90% of periodontal pathogens and the results showed little or no effect on the cells for the parameters studied. This implies minimal cellular damage with topical application of these antibiotics; although, further work needs to be done before these findings can be extrapolated to *in vivo* conditions.

The tetracyclines are particularly versatile in their ability to combat oxidative stress, mop up free radicals and inhibit an excessive inflammatory response secondary to an antigenic stimulus [29]. These features of inflammation characterise some of the more aggressive forms of early onset periodontal diseases. The actions of tetracyclines mentioned above have been utilised in the evolution of non-antimicrobial chemically modified tetracyclines (CMTs) and low dose doxycycline, with the specific aim of curtailing the damaging effects of an over-exuberant inflammatory response seen in certain categories of periodontal diseases, often compounded by poorly controlled diabetes mellitus and cardiovascular disease. In the management of these cases presenting with periodontal disease the emphasis would be on thorough root surface debridement to reduce the microbial presence to sub-pathogenic levels and the adjunctive use of non-antimicrobial tetracyclines / low dose doxycycline formulations to modulate an excessive host response that can be damaging. Some of the positive clinical outcomes when using these formulations for the adjunctive management of periodontal patients, are due to the non-antimicrobial anti-inflammatory actions of this group of drugs in an over-exuberant inflammatory milieu that is damaging rather than protective.

## APPLICATIONS FOR NON-ANTIMICROBIAL ACTIONS OF TETRACYCLINES IN PERIODONTAL DISEASES AND DIABETES MELLITUS (DM)

Oxidative stress is a unifying mechanism for production of reactive oxygen species and plays a significant role in the manifestation of insulin resistance, atherogenic dyslipidaemia [30] and periodontal disease [4]. The term metabolic syndrome is used to describe a clustering of risk factors of metabolic origin for cardiovascular disease and type 2 diabetes. These include hyperglycaemia, hypertension, dyslipidaemia and a pro-inflammatory state often associated with obesity [31]. It is relevant that patients with metabolic syndrome show significant correlation with the prevalence of severe periodontal disease initiated by pathogenic bacterial plaque. This is associated with high levels of inflammatory cytokines, other markers of inflammation and oxidative stress such as C-reactive proteins and low density lipoproteins, on par with metabolic diseases [32]. Periodontal patients with co-existing features of metabolic syndrome constitute a good model for therapeutic interventions which result in improved metabolic control of their systemic diseases [33]. The non-antimicrobial anti-inflammatory actions of tetracyclines have useful applications in the management of inflammatory diseases as detailed below.

Early in the course of diabetes mellitus (DM), mRNAs for IL-1β, TNF-α and other pro-inflammatory mediators are increased in the retina, partly from activated microglia. Minocycline inhibits diabetes-induced cytokine and cytotoxin production and holds promise in preventing retinal complications of DM [34]. Bacterial LPS also causes marked upregulation and release of IL-1β, TNF-α and NO in retinal microglia; this was inhibited by minocycline which has significant impact in reducing the expression and release of these mediators [35]. Recent investigations [36] have demonstrated that doxycycline was more effective in inhibiting matrix metallo proteinases (MMPs) in human aortic smooth muscle cells than minocycline, by upregulating the MMP inhibitor TIMP-1 (Tissue inhibitor of metallo proteinase-1). These findings have implications on periodontal diseases initiated by lipopolysaccharide mediated inflammatory burden with applications for the MMP inhibitory actions of tetracyclines.

Doxycycline hyclate has been shown to accelerate periodontal wound healing in diabetic mice [37] and humans [38]. Similar studies with Arestin (minocycline microspheres) have shown reduction in HbA1c and improved periodontal disease control over root debridement alone [39]. Adjunctive locally delivered doxycycline in periodontal pockets of smokers has been shown to be more effective than pocket debridement alone [40] in reducing the parameters of inflammatory periodontal disease. A combination of alendronate and low dose doxycycline has demonstrated improved bone remodelling and decreased rate of progression of experimental periodontits in rats [41]. Both minocycline and doxycycline cause significant stimulation of osteoblastic cells at levels conventionally detected in plasma and gingival crevicular fluid; long term exposure of these cells to tetracyclines resulted in a proportional increase in mineralised bone matrix; while exposure to higher levels of these drugs resulted in delayed cell proliferation and differentiation [42]. The above studies have implications on healing responses using adjunctive teracyclines in the management of periodontal diseases in diabetic patients who smoke. This can overcome damaging effects of the oxidative stress imposed.

### Oxidative Stress Induced Sequelae of Diabetes, Dislipidaemia and Cardiovascular Disease: Context of Therapeutic Intervention with Tetracyclines as Antioxidants

Inflammatory burden from periodontal disease could compound the complications of diabetes mellitus and cardiovascular disease in a systemically compromised population. Fluctuating post-prandial glucose levels associated with higher levels of cytokines during hyperglycaemic spikes, contribute to oxidative stress induced damage more so than chronically elevated levels [43, 44]. A nutritional overload resulting in excessive amounts of glucose and fatty acids leads to ROS production. Interactions between glucose and plasma proteins result in the formation of advanced glycation end products (AGE) which in turn initiate the production of TNF-α, IL-6, IL-18 and ROS; this contributes to chemical modification of lipoproteins and atherogenesis [45]. Administration of the antioxidant glutathione completely suppressed elevation of cytokines in response to glucose pulses. This implies that an oxidative stress induced mechanism is responsible for the inflammatory burden imposed by hyperglycaemia in humans [46] with implications on atherosclerosis and cardiovascular disease. The oxidative stress induced pathogenesis of periodontal diseases is not dissimilar and has applications for the adjunctive use of tetracyclines as antioxidants [47, 48] in the management of systemically compromised periodontal patients.

The above correlations seen in metabolic syndrome are often linked to obesity which has reached pandemic proportions. Abdominal or visceral fat associated with waist circumference has a stronger correlation as a risk factor than peripheral fat [49, 50].****The combination of cigarette smoking and metabolic syndrome is particularly damaging. Smoking cessation should be emphasised for all patients with diabetes, cardiovascular disease and periodontal diseases. The UK guidelines on smoking cessation suggest a specific plan that incorporates a holistic approach to integrate educational, behavioural and pharmacological components to aid cessation and prevent relapse [51].

The pathophysiology of diabetes mellitus and cardiovascular disease discussed above has implications on the progression of periodontal diseases. These risk criteria for oxidative stress induced tissue damage have a logical place in the context of progressive loss of periodontal support for teeth. The relationship between periodontitis and diabetes is reciprocal [52, 53], for example AGE related cytokine spikes are of relevance in the pathogenesis of periodontal diseases and vice versa considering the importance of an inflammatory burden, oxidative stress and its sequelae for the progression of both disease entities.

## SPECIFIC ANTI-INFLAMMATORY AND ANTI-ANGIOGENIC TARGETS OF TETRACYCLINES

The collagenase MMPs that breakdown collagen are MMP-1, 8 and13 and those that affect basement membrane collagen (collagen IV) are the gelatinases known as MMPs 2 and 9. Tetracycline and its analogues inhibit these enzyme systems [54].

Angiogenesis is facilitated by matrix degrading enzymes such as MMPs.

Minocycline and doxycycline have been shown to inhibit angiogenesis by preventing endothelial growth and activity of collagenase [55]. Inhibition of synthesis of MMP-8 and MMP-9 by endothelial cells in response to doxycycline and to a lesser extent by the chemically modified tetracyclines (CMTs) has been demonstrated at the mRNA level [56]. These effects of tetracyclines have therapeutic implications on inflammatory processes associated with vascular tissue development.

Elastin degradation and MMP activity are reduced by doxycycline in a model representing aneurismal disease [57]. In a cell culture model of corneal epithelial cells treated with lipopolysaccharide, doxycycline inhibited the degree of formation of IL-1β to an extent that was similar to that of corticosteroids [58]. It also prevents endotoxemia *in vivo* [59]. Doxycycline can cause dose dependent reduction in the production of the cytokines IL-1β, IL-6, TNF-α and IFN-γ [60].

Matrix metallo proteinases mediate different physiological processes by digesting extracellular matrix components. The pathogenesis of several diseases is characterised by over-expression of MMPs. The activity of MMPs is regulated by tissue inhibitors of MMPs (TIMPs) found in bone and other cells. Their biosynthesis is regulated by local and systemic hormones, uptake and degradation by cells. Considering their actions, abnormal expression of MMPs may lead to pathological conditions affecting bone and cartilage. Pharmacological agents that function as inhibitors of matrix metalloproteinases have useful therapeutic potential [61].

## THERAPEUTIC ADVANTAGES OF MATRIX METALLO PROTEINASE INHIBITION

Subantimicrobial dosing with doxycycline (SDD; 20mg bd) has been shown to consistently reduce the activity of mammalian collagenase and other matrix metalloproteinases in the tissues of the periodontium. A recent study on 19 periodontal patients scheduled for flap surgery demonstrated that short term therapy with SDD alone reduced levels of proteolytic enzymes, while low dose flurbiprofen alone had no significant effect; combined medication with both agents resulted in statistically significant synergistic reduction in collagenase, gelatinase, serpinolytic activities and to a lesser extent, elastase activity [62]. Previous animal studies suggest a mechanism whereby NSAIDs contribute to increased uptake of the tetracycline compound in inflammatory tissue, resulting in a synergistic effect and enhanced therapeutic impact. Other studies reinforce the efficacy of subantimicrobial dosing with doxycycline [63] in smokers and non-smokers compared with root surface debridement alone in significantly reducing periodontal disease parameters [64].

An increase in MMPs associated with equine recurrent airway obstruction was effectively inhibited by chemically modified tetracyclines, particularly CMT-3, being significantly more effective than biphosphonates against some proteases [65]. Definitive MMP inhibitors have potential value in overcoming excessive tissue destruction associated with proteinases in chronic lung disease and the advantage of having minimal side effects over other agents. Acute respiratory distress syndrome can be the cause of death in cases of multiple organ failure. A chemically modified tetracycline was shown to inhibit the development of acute respiratory distress and also prevent septic shock in a slow onset porcine model by inhibiting numerous inflammatory mediators [66]. These actions have clinical applications in curbing an over-exuberant inflammatory response in the pathogenesis of periodontal diseases.

## CARDIOPROTECTIVE EFFECTS OF TETRACYCLINES

In a murine model administration of 4mg/kg tetracycline dramatically reduced the size of infarct area in a murine heart analysed by tetrazolium staining [67]. Translational inhibition in mitochondria resulting in cold stress associated molecules may be a cardioprotective effect of tetracyclines. A subclinical dose of tetracycline is likely to protect the heart from ischemic injury and be of therapeutic value in suppressing the onset of infarction caused by myocardial ischaemia [67]; this demonstrates another application for subclinical dosing with tetracycline, also used in the adjunctive management of periodontal disease of potential value in those presenting with coexisting cardiovascular disease .

Myocardial ischaemia is associated with the activation of MMPs and the serine proteinase plasmin. In a rat model, pre-treatment with Doxycycline has been shown to reduce myocardial infarct size by 37% [68]. Doxycycline inhibits the serine protease plasmin while treatment with a broad spectrum MMP inhibitor had no effect. Inhibition of plasmin by doxycycline may reduce myocyte death and contribute to cardioprotection. The effects of timing of this intervention was investigated in a rat myocardial infarct model in comparison with methyl prednisolone and an aqueous vehicle at early (24h) and late (2-7days) post-myocardial infarction [69]. The doxycycline group had an improved outcome for myocardial recovery compared with methyl prednisolone. Inhibition of the serine protease plasmin appears to have therapeutic advantage in myocardial infarct patients.

## WOUND HEALING ACTIONS OF TETRACYCLINES

Modulation of basement membrane laminin, MMPs, osteoblast and osteoclast functions by tetracyclines contribute to its effects on wound healing. The effect of low dose tetracycline on modulation of laminin-5 and its association with proliferation of junctional epithelial cells during pocket formation was investigated in 30 patients with chronic periodontal disease. Root surface debridement was performed with or without adjunctive low dose doxycycline for its non-antimicrobial effects [70] and monitored 3 monthly for twelve months. Gingival crevicular fluid (GCF) samples were collected for analysis of laminin and clinical paramenters of periodontal disease were recorded. The test group of patients with periodontal disease was subjected to complete root surface debridement with 20mg bd of low dose tetracycline and compared with the randomly selected control group who received root surface debridement plus placebo only. It is relevant that the low dose doxycycline group showed a significant reduction in GCF levels of Laminin-5 gamma 2 chain fragments compared with the placebo group. Matrix metallo proteinase mediated fragmentation of laminin-5 can contribute to pocket formation by stimulating epithelial cell migration. Reducing these levels, could be another mechanism whereby low dose doxycycline could contribute to resolution of periodontal pockets in the long-term.

Chemically modified tetracyclines are effective in inhibiting bone resorption by inhibiting osteoclastic actions and inducing apoptosis of osteoclasts, in addition to reducing bone resorption by inhibiting matrix metallo proteinases. Recent studies [71] demonstrate that the non-antibiotic analogues CMT-3 and CMT-8 of doxycycline and minocycline respectively are potent inhibitors of osteoclastogenesis from peripheral blood monocytes in response to macrophage colony stimulating factor and RANK at a concentration of 250ng/ml. The mechanism is reported to be independent of osteoblast osteoclast interactions. Over a 24h period, both CMT-3 and CMT-8 also induced apoptosis of mature osteoclasts at concentrations of 5-20 μg/ml. Inhibition of osteoclastogenesis and induction of osteoclast apoptosis are significant actions of CMTs, independent of their matrix metalloproteinase inhibitory action or osteoblastic interaction [71].

It has been shown that CMT-8 and oestrogen can improve wound healing in ovariectomised rats by changing the quality of wound bed collagen with improved expression of key molecules in wound healing such as laminin-5 gamma 2-chain [72]. This illustrates another mechanism whereby non-antimicrobial tetracyclines can contribute to healing responses. A randomized, placebo controlled double masked study compared the effects of administration of low dose doxycycline to periodontal patients with or without associated flap surgery [73], using clinical parameters, microbial and bone markers. Adjunctive low dose doxycycline was shown to improve periodontal healing and reduce local bone resorption. These findings reinforce results of other workers in confirming that tetracyclines and their derivatives have potential therapeutic benefits in the management of metabolic diseases affecting bone homeostasis by modulating osteoblast and osteoclast activities. A novel finding regarding the inhibition of IL-1β induced IL-6 expression by CMT-8 in murine osteoblasts at a post-transcriptional level affecting the stability of IL-6 mRNA, provides potential for new incentives for therapeutic management of IL-6 mediated metabolic bone diseases [74].

## ANTI-APOPTOTIC ACTIONS OF TETRACYCLINES

Several studies demonstrate anti-apoptotic and anti-inflammatory effects of minocycline in the context of neuro- protection [75-78] and in a lung epithelial cell model [79]. In the murine lung alveolar epithelial cell model [79] treated with cytomix (TNF-α, IL-1β, IFN-gamma each at 5ng/ml), doxycycline was effective in reducing the formation of nitric oxide (NO) from inducible nitric oxide synthase (iNOS), by reducing the expression of p38 MAPK resulting in destabilization of iNOS mRNA. In a rat model marked inflammation was induced in the striatum by injecting quinolinic acid resulting in enhanced expression of iNOS and cyclooxygenase-2 [78]. These responses were reduced with administration of either minocycline or pyruvate an end product of glycolysis which could direct metabolism along a less damaging protective pathway; combined administration of both agents resulted in significant attenuation of the inflammatory response compared with either agent alone. This has implications on a therapeutic approach for neurodegenerative disorders with excitotoxic insults and may also have applications for other diseases which manifest oxidative stress induced tissue damage such as periodontal disease, diabetes and cardiovascular diseases.

When cell free assays of rat brain homogenate were done to test the antioxidant potency of minocycline it demonstrated direct antioxidant effects with radical scavenging activity comparable with α-tocopherol, a synthetic, racemic vitamin E [75]. The radical scavenging actions of minocycline are consistent with its multi-substituted phenol ring similar to that of vitamin E, belonging to the class of phenolic antioxidants; they are effective in scavenging free radicals resulting in the formation of phenol derived free radicals which are relatively stable and non-reactive. Minocycline was found to be far more potent than tetracycline in its radical scavenging potency and inhibition of lipid peroxidation by 316- and 200-fold respectively. Minocycline HCl has been shown to be very effective in quenching H_2_O_2_ levels; relative rates of various ROS related processes could contribute to a combination of quenching and scavenging of ROS by tetracyclines [80]. These actions of minocycline could explain its potency in reducing the parameters of periodontal disease progression during adjunctive usage and cardioprotection in reducing the size of myocardial infarcts [68].

ROS are implicated in periodontal tissue damage seen during the progression of inflammatory periodontal diseases. A net damaging outcome is likely to be seen with an imbalance in oxidant /antioxidant activity due to inadequate protection from antioxidants such as glutathione. A study on periodontal patients demonstrated that resolution of periodontal disease parameters resulted in a significant decrease in lipid peroxidation and an increase in salivary glutathione levels [81]. Products of lipid peroxidation contribute to periodontal disease progression. Accumulation of lipid peroxidase products leading to raised levels of oxidative stress and imbalance of endogenous antioxidant defence at inflammatory sites, has been reported in periodontal patients by other workers [82, 83]. ROS activity has been shown to be significantly greater in chronic periodontitis than in non-periodontitis subjects [84]. In this context the antioxidant and radical scavenging properties of minocycline are particularly relevant in the adjunctive management of periodontal diseases with or without additional inflammatory burden from co-existing systemic diseases.

## SUMMARY AND CONCLUSIONS

Periodontal patients often present with multiple medication for DM, CVD arthritis and other systemic diseases. The presence of a significant inflammatory burden is a common feature driving these conditions with therapeutic applications for using adjunctive tetracyclines in a non anti-microbial capacity. These actions of tetracyclines encompassing matrix metalloproteinase inhibition, antioxidant and anabolic effects have been addressed in addition to their antimicrobial applications in the adjunctive management of periodontal diseases. Some of the positive outcomes of periodontal healing in response to tetracyclines as antimicrobial adjuncts could be attributed to their anti-inflammatory, antioxidant and matrix stimulatory actions. For instance a study on periodontal patients with Type 1 diabetes mellitus demonstrated that adjunctive topical application of doxycycline hyclate in addition to root surface debridement resulted in statistically significant improvement in parameters of periodontal healing at 12 months [85].

In a rat model of induced periodontal disease using gingival LPS injections, elevated levels of collagenase (MMP-8), gelatinase (MMP-9), elastase and alveolar bone loss were overcome by using a synergistic combination of sub-optimal doses of the chemically modified tetracycline CMT-8 and a biphosphonate, clodronate [86]; when sub-optimal doses of these agents were used individually, there were negligible effects on the above parameters. This synergy has therapeutic potential in preventing periodontal tissue damage in patients with metabolic and other inflammatory diseases such as arthritis. The pathophysiology of periodontal diseases and arthritis is similar [87]; the confounding effects of medication has made it difficult to establish a clear link. In a recent study of adjuvant induced arthritis in a rat model, arthritic changes were associated with raised levels of MMPs, IL-1β and TNF-α in joint tissue; this was ameliorated after TIMP-4 gene therapy in the treated group compared with the untreated control animals [88]. These findings confirm the relevance of an over-exuberant inflammatory response in the pathogenesis of arthritis, in common with periodontal and metabolic diseases; reinforcing the non-antimicrobial therapeutic role of tetracyclines in controlling an excessive inflammatory burden in view of their MMP inhibitory actions.

Adjunctive use of tetracyclines for their non-antimicrobial effects can aid in overcoming the secondary effects of diabetes, dyslipidaemia and cardiovascular diseases in periodontal patients, particularly those who smoke. Reduction in the parameters of oxidative stress contribute to reduced inflammatory burden; this can contribute towards minimising the likelihood of overt clinical presentation of these diseases in subclinical cases or act in a therapeutic capacity in those with overt disease. Specific mechanisms of action of this group of drugs has been discussed.

It is relevant that other agents with anti-inflammatory, antioxidant and pro-anabolic effects have been identified over the last decade and shown promise in controlling over-exuberant inflammatory responses with associated oxidative stress induced tissue damage applicable to the disease entities discussed. Macrophages activated by bacterial components release ROS and reactive nitrogen species (RNS), a critical part of defence mechanisms. Grape seed proanthocyanidins demonstrate a spectrum of actions against oxidative stress. The effect of this compound on murine macrophages stimulated with lipopolysaccharides derived from periodontopathogens showed significant reduction in NO, ROS production and inducible nitric oxide synthase expression [89], demonstrating strong antioxidant properties of proanthocyanidins with potential therapeutic applications for periodontal and other inflammatory diseases. An investigation of the relationship between serum levels of the antioxidant lycopene, linked to monthly tomato consumption and congestive cardiac failure in periodontitis patients, showed a significant positive correlation between serum lycopene levels and cardioprotection; with an inverse relationship between serum lycopene and C-reactive protein [90].

Recently Ginkgo biloba (Gb) extracts have been shown to have antioxidant and anti-inflammatory properties in reducing ROS levels. They consist of flavonoids and terpenoids. EGb 761, a standard extract of Gb was used on the model Caenorhabditis elegans when subjected to thermal stress. There was attenuation of ROS accumulation in response to the Gb extract, reduction in the transcription of stress inducible catalase genes and glutathione S-transferase 4, demonstrating its ability to combat oxidative stress [91]. In another study, mercury toxicity induced in a rat model demonstrated oxidative stress with raised levels of malondialdehyde, myeloperoxidase and reduced levels of glutathione in multiple organs. In the animals treated with Gb extract there was significant reversal in levels of the above markers of oxidative stress confirming the antioxidant effects of Gb [92].

In a diabetogenic cell culture model, we have demonstrated the antioxidant efficacy of glutathione and insulin like growth factor in overcoming oxidative stress induced effects of glucose, AGE and nicotine [93]. The antioxidants co-enzyme Q10, phytoestrogens and Pycnogenol derived from French maritime pine bark have demonstrated effective amelioration of oxidative stress in a cell culture model applicable to periodontal disease in smokers [94]. These studies highlight the importance of oxidative stress induced disease mechanisms and the relevance of combating the damaging effects of reactive oxygen species for effective control of periodontal and systemic diseases.

The anti-inflammatory effects of the established antioxidant agents described above in the context of periodontal and metabolic diseases have potential for therapeutic use with non-antimicrobial formulations of tetracyclines in suboptimal doses to minimise side effects. The non-antimicrobial actions of tetracyclines have shown remarkably similar mechanisms to those of agents with established anti-inflammatory / antioxidant potential. These findings clarify the multi-faceted actions of tetracyclines which are unique amongst antimicrobials, with therapeutic applications in periodontal and metabolic diseases.

## References

[1] Georgiou TO, Marshall RI, Bartold PM (2004). Prevalence of systemic diseases in Brisbane general and periodontal practice patients. Aust Dent J.

[2] Molloy J, Wolff LF, Lopez-Guzman A, Hodges JS (2004). The association of periodontal disease parameters with systemic medical conditions and tobacco use. J Clin Periodontol.

[3] Soory M (2002). Hormone mediation of immune responses in the progression of diabetes, rheumatoid arthritis and periodontal diseases. Curr Drug Targets-Immun, Endocr Metab Disord.

[4] Soory M (2004). Biomarkers of diabetes mellitus and rheumatoid arthritis associated with oxidative stress, applicable to periodontal diseases. Curr Top Steroid Res.

[5] Tsai C, Hayes C, Taylor GW (2002). Glycaemic control of Type 2 diabetes and severe periodontal disease in the US adult population. Community Dent Oral.

[6] Socransky S, Haffajee A (1991). Microbial mechanisms in the pathogenesis of destructive periodontal diseases a critical assessment. J Periodont Res.

[7] Page RC (1998). The pathobiology of periodontal diseases may affect systemic diseases inversion of a paradigm. Ann Periodontol.

[8] Chun YH, Chun KR, Olguin D, Wang HL (2005). Biological foundation for periodontitis as a potential risk factor for atherosclerosis. J Periodont Res.

[9] Bergstrom J (2004). Tobacco smoking and chronic destructive disease. Odontol.

[10] Scott DA, Palmer RM, Stapleton JA (2001). Validation of smoking status in clinical research into inflammatory periodontal disease. J Clin Periodontol.

[11] Holmstrup P, Poulsen AH, Andersen L, Skuldbol T, Fiehn NE (2003). Oral infections and systemic diseases. Dent Clin North Am.

[12] Taylor GW (1999). Periodontal treatment and its effects on glycaemic control: a review of the evidence. Oral Surg Oral Med Oral Pathol Oral Radiol Endod.

[13] Deliargyris EN, Madianos PN, Kadoma W (2004). Periodontal disease in patients with acute myocardial infarction prevalence and contribution to elevated C-reactive protein levels. Am Heart J.

[14] Montebugnoli L, Servidio D, Miaton RA (2005). Periodontal health improves systemic inflammatory and haemostatic status in subjects with coronary heart disease. J Clin Periodontol.

[15] Beck JD, Eke P, Heiss G, Madianos P (2005). Periodontal disease and coronary heart disease a reappraisal of the exposure. Circulation.

[16] Kirkwood KL, Cirelli JA, Rogers JE, Giannobile WV (2007). Novel host response therapeutic approaches to treat periodontal diseases. Periodontology 2000.

[17] Sapadin AN, Fleischmajer R (2006). Tetracyclines Nonantibiotic properties and their implications. J Am Acad Dermatol.

[18] Nelson ML (1998). Chemical and biological dynamics of tetracyclines. Adv Dent Res.

[19] Kaner D, Bernimoulin JP, Hopfenmuller W, Kleber BM, Friedmann A (2007). Controlled delivery chlorhexidine chip versus amoxicillin/ metronidazole as adjunctive antimicrobial therapy for generalised aggressive periodontitis a randomised controlled clinical trial. J Clin Periodontol.

[20] Horz HP, Conrads G (2007). Diagnosis and anti-infective therapy of periodontitis. Expert Rev Anti infect Ther.

[21] Mascarenhas P, Gapski R, Al-Shammari K (2005). Clinical response of azithromycin as an adjunct to non-surgical periodontal therapy in smokers. J Periodontol.

[22] Grossi SG, Goodson JM, Gunsolley JC (2007). Mechanical therapy with adjunctive minocycline microspheres reduces red complex bacteria in smokers. J Periodontol.

[23] Hanes PJ, Purvis JP (2003). Local anti-infective therapy Pharmacological agents. A systematic review. Ann Periodontol.

[24] Paquette DW, Hanlon A, Lessem J, Williams RC (2004). Clinical relevance of adjunctive minocycline microspheres in patients with chronic periodontitis: secondary analysis of a phase 3 trial. J Periodontol.

[25] Lu H-K, Chei C-J (2005). Efficacy of subgingivally applied minocycline in the treatment of chronic periodontitis. J Periodont Res.

[26] Persson RG, Salvi GE, Heitz-Mayfield LJA, Lang NP (2006). Antimicrobial therapy using a local drug delivery system Arestin in the treatment of peri-implantitis. 1: microbiological outcomes. Clin Oral Implants Res.

[27] Heaton PC, Fenwick SR, Brewer DE (2007). Association between tetracycline or doxycycline and hepatotoxicity a population based case-control study. J Clin Pharm Ther.

[28] Suzuki A, Yagisawa J, Kumakura S, Tsutsui T (2006). Effects of minocycline and doxycycline on cell survival and gene expression in human gingival and periodontal ligament cells. J Periodont Res.

[29] Webster G, Del Rosso JQ (2007). Anti-inflammatory activity of tetracyclines. Dermatol Clin.

[30] Wright Jr E, Scism-Bacon JL (2006). Glass LC 2006. Oxidative stress in type 2 diabetes the role of fasting and postprandial glycaemia. Int J Clin Pract.

[31] Grundy SM (2006). Does a diagnosis of metabolic syndrome have value in clinical practice. Am J Clin Nutr.

[32] Nishimura F, Murayama Y (2001). Periodontal inflammation and insulin resistance - lessons from obesity. J Dent Res.

[33] Promsudthi A, Pimapansri S, Deerochanawong C, Kanchanavasita W (2005). The effect of periodontal therapy on uncontrolled type 2 diabetes mellitus in older subjects. Oral disease.

[34] Krady JK, Basu A, allen CM, Xu Y, LaNoue KF, Gardner TW, Levison SW (2005). Minocycline reduces proinflammatory cytokine expression2005. microglial activation and caspase-3 activation in a rodent model of diabetic retinopathy. Diabetes.

[35] Wang AL, Yu AC, Lau LT (2005). Minocycline inhibits LPS-induced retinal microglia activation. Neurochem Int.

[36] Yao JS, Shen F, Young WL, Yang GY (2007). Comparison of doxycycline and minocycline in the inhibition of VEGF-induced smooth muscle cell migration. Neurochem Int.

[37] Kol R, Palattella A (2006). The use of doxycycline in periodontology. Histologic *in vivo* study on mice affected by diabetes mellitus. Minerva Stomatol.

[38] Llambes F, Silvestre FJ, Hernandez-Mijares A, Guiha R, Caffesse R (2005). Effect of non-surgical periodontal treatment with or without doxycycline on the periodontium of type 1 diabetic patients. J Clin Periodontol.

[39] Skaleric U, Schara R, Medvescek M, Hanlon A, Doherty F, Lessem J (2004). Periodontal treatment by Arestin and its effects on glycaemic control in Type 1 diabetic patients. J Int Acad Periodontol.

[40] Machion L, Andia DC, Lecio G (2006). Locally delivered doxycycline as an adjunctive therapy to scaling and root planing in the treatment of smokers a 2-year follow up. J Periodontol.

[41] Buduneli E, Buduneli N, Vardar-Sengul S, Kardesler L, Atilla G, Lappin D, Kinane DF (2005). Systemic low-dose doxycycline and alendronate administration and serum interleukin-1 beta, osteocalcin and C-reactive protein levels in rats. J Periodontol.

[42] Gomes PS, Fernandes MH (2007). Effect of therapeutic levels of doxycycline and minocycline in the proliferation and differentiation of human bone marrow osteoblastic cells. Arch Oral Biol.

[43] Hirsh IB, Brownlee M (2005). Should minimal blood glucose variability become the gold standard of glycaemic control?. J Diabetes Complicat.

[44] Hirsh IB (2005). Intensifying insulin therapy in patients with Type 2 diabetes mellitus. Am J Med.

[45] Basta G, Schmidt AM, DeCaterina R (2004). Advanced glycation end products and vascular inflammation implications for accelerated atherosclerosis in diabetes. Cardiovasc Res.

[46] Esposito K, Nappo F, Marfella R (2002). Inflammatory cytokine concentrations are acutely increased by hyperglycaemia in humans role of oxidative stress. Circulation.

[47] Kelly KJ, Sutton TA, Weathered N (2004). Minocycline inhibits apoptosis and inflammation in a rat model of ischaemic renal injury. Am J Physiol Renal Physiol.

[48] Jordan J, Fernandez-Gomez FJ, Ramos M, Ikuta I, Aguirre N, Galindo MF (2007). Minocycline and cytoprotection shedding new light on a shadowy controversy. Curr Drug Deliv.

[49] Ritchie CS (2007). Obesity and periodontal disease. Periodontology 2000.

[50] Saito T, Shimazaki Y (2007). Metabolic disorders related to obesity and periodontal disease. Periodontology 2000.

[51] West R, McNeill A, Raw M (2000). Smoking cessation guidelines for health professionals an update. Health Education Authority. Thorax.

[52] Grossi SG, Genco RJ (1998). Periodontal disease and diabetes mellitus a two-way relationship. Ann Periodontol.

[53] Mealey BL, Ocampo GL (2007). Diabetes mellitus and periodontal disease. Periodontology 2000.

[54] Golub LM, Lee HM, Ryan ME, Giannobile WV, Payne J, Sorsa T (1998). Tetracyclines inhibit connective tissue breakdown by multiple non-antimicrobial mechanisms. Adv Dent Res.

[55] Guerin C, Laterra J, Masnyk T, Golub LM, Brem H (1992). Selective endothelial growth inhibition by tetracyclines that inhibit collagenase. Biochem Biophys Res Commun.

[56] Hanemaaijer R, Visser H, Koolwijk P (1998). Inhibition of MMP synthesis by doxycycline and chemically modified tetracyclines in human endothelial cells. Adv Dent Res.

[57] Boyle JR, McDermott E, Crowther M, Wills AD, Bell PR, Thompson MM (1998). Doxycycline inhibits elastin degradation and reduces metalloproteinase activity in a model of aneurismal disease. J Vasc Surg.

[58] Solomon A, Rosenblatt M, Li DQ (2000). Doxycycline inhibition of interleukin-1 in the corneal epithelium. Invest Ophth Vis Sci.

[59] Milano S, Arcoleo F, D'Agostino P, Cillari E (1997). Intraperitoneal injection of tetracyclines protects mice from lethal endotoxemia downregulating inducible nitric oxide synthase in various organs and cytokine nitrate secretion in blood. Antimicrob Agents Ch.

[60] Krakauer T, Buckley M (2003). Doxycycline is anti-inflammatory and inhibits Staphylococcal exotoxin-indued cytokines and chemokines. Anitimicrob Agents Chemother.

[61] Varghese S (2006). Matrix metalloproteinases and their inhibitors in bone an overview of regulation and functions. Front Biosci.

[62] Lee HM, Ciancio SG, Tuter G, Ryan ME, Komaroff E, Golub M (2004). Subantimicrobial dose doxycycline efficacy as a matrix metalloproteinase inhibitor in chronic periodontitis patients is enhanced when combined with a non-steroidal anti-inflammatory drug. J Periodontol.

[63] Salvi GE, Lang NP (2005). Host response modulation in the management of periodontal diseases. J Clin Periodontol.

[64] Preshaw PM, Hefti AF, Bradshaw MH (2005). Adjunctive subantimicrobial dose doxycycline in smokers and non-smokers with chronic periodontitis. J Clin Periodontol.

[65] Raulo SM, Sorsa T, Maisi P (2006). *In vitro* inhibition of matrixmetallo-proteinase activity in tracheal epithelial lining fluid from horses with recurrent airway obstruction. Am J Vet Res.

[66] Steinberg J, Halter J, Schiller H (2005). Chemically modified tetracycline prevents the development of septic shock and acute respiratory distress syndrome in a chemically applicable porcine model. Shock.

[67] Kagawa N, Senbonmatsu TA, Satoh K (2005). Tetracycline protects myocardium against ischaemic injury. Front Biosci.

[68] Griffin MO, Jinno M, Miles LA, Villarreal FJ (2005). Reduction of myocardial infarct size by doxycycline a role for plasmin inhibition. Mol Cell Biochem.

[69] Garcia RA, Go KV, Villarreal FJ (2007). Effects of timed administration of doxycycline or methylprednisolone on post-myocardial infarction inflammation and left ventricular remodelling in the rat heart. Mol Cell Biochem.

[70] Emingil G, Atilla G, Sorsa T, Savolainen P, Baylas H (2004). Effectiveness of adjunctive low-dose doxycycline therapy on clinical parameters and gingival crevicular fluid laminin-5 gamma2 chain levels in chronic periodontitis. J Periodontol.

[71] Holmes SG, Still K, Buttle DJ, Bishop NJ, Grabowski PS (2004). Chemically modified tetracyclines act through multiple mechanisms directly on osteoclast precursors. Bone.

[72] Pirila E, Parikka M, Ramamurthy NS (2002). Chemically modified tetracycline (CMT-8) and oestrogen promote wound healing in ovariectomised rats effects on matrix metalloproteinase-2 membrane type1 matrixmetalloproteinase, and laminin-5 gamma2-chain. Wound Repair Regen.

[73] Gapski R, Barr JL, Sarment DP, Layher MG, Socransky SS, Giannobile WV (2004). Effect of systemic matrix metalloproteinase inhibition on periodontal wound repair a proof of concept trial. J Periodontol.

[74] Kirkwood K, Martin T, Andreadis ST, Kim YJ (2003). Chemically modified tetracyclines selectively inhibit IL-6 expression in osteoblasts by decreasing mRNA stability. Biochem Pharmacol.

[75] Kraus RL, Pasieczny R, Lariosa-Willingham K, Turner MS, Jiang A, Trauger JW (2005). Antioxidant properties of minocycline neuroprotection in an oxidative stress assay and direct radical-scavenging activity. J Neurochem.

[76] Xu L, Fagan SC, Waller JL (2004). Low dose intravenous minocycline is neuroprotective after middle cerebral artery occlusion-reperfusion in rats. BMC Neurol.

[77] Stirling DP, Khodarahmi K, Liu J (2004). Minocycline treatment reduces delayed oligodendrocycte death, attenuates axonal dieback and improves functional outcome after spinal cord injury. J Neurosci.

[78] Ryu JK, Choi HB, McLarnon JG (2006). Combined minocycline plus pyruvate treatment enhances effects of each agent to inhibit inflammation, oxidative damage and neuronal loss in an excitotoxic animal model of Huntington's disease. Neuroscience.

[79] Hoyt JC, Ballering J, Numanami H, Hayden JM, Robbins RA (2006). Doxycycline modulates nitric oxide production in murine lung epithelial cells. J Immunol.

[80] Miyachi Y, Yoshioka A, Imamura S, Niwa Y (1986). Effect of antibiotics on the generation of reactive oxygen species. J Invest Dermatol.

[81] Tsai CC, Chen HS, Chen SL (2005). Lipid peroxidation: a possible role in the induction and progression of chronic periodontitis. J Periodont Res.

[82] Krol K (2004). Reactive oxygen species and antioxidant mechanisms in the pathogenesis of periodontitis. Ann Acad Med Stetin.

[83] Panjamurthy K, Manoharan S, Ramachandran CR (2005). Lipid peroxidation and antioxidant status in patients with periodontitis. Cell Mol Biol Lett.

[84] Chapple ILC (2006). Oxidative stress, nutrition and neutrogenomics in periodontal health and disease. Int J Dent Hyg.

[85] Martorelli de Lima AF, Cury CC, Palioto DB, Duro AM, da Silva RC, Wolff LF (2004). Therapy with adjunctive doxycycline local delivery in patients with type 1 diabetes mellitus and periodontitis. J Clin Periodontol.

[86] Llavaneras A, Ramamurthy NS, Heikkila P (2001). A combination of a chemically modified doxycycline and a biphosphonate synergistically inhibits endotoxin-induced periodontal breakdown in rats. J Periodontol.

[87] Soory M (2007). Periodontal diseases and rheumatoid arthritis. A coincident model for therapeutic intervention?. Curr Drug Metab.

[88] Ramamurthy NS, Greenwald RA, Celiker MY, Shi EY (2005). Experimental arthritis in rats induces biomarkers of periodontitis which are ameliorated by gene therapy with tissue inhibitor of matrix metalloproteinases. J Periodontol.

[89] Houde V, Grenier D, Chandad F (2006). protective effects of grape seed proanthocyanidins against oxidative stress induced by lipopolysaccharides of periodontopathogens. J Periodontol.

[90] Wood N, Johnson RB (2004). The relationship between tomato intake and congestive heart failure risk in periodontitis subjects. J Clin Periodontol.

[91] Kampkotter A, Pielarski T, Rohrig R (2007). The Ginkgo biloba extract EGb761 reduces stress sensitivity, ROS accumulation and expression of catalase and glutathione S-transferase 4 in Caenorhabditis elegans. Pharmacol Res.

[92] Sener G, Sehirli O, Tozan A, Velioglu-Ovunc A, Gedik N, Omurtag GZ (2007). Ginkgo biloba extract protects against mercury (ll)-induced oxidative tissue damage in rats. Food Chem Toxicol.

[93] Rahman ZA, Soory M (2006). Antioxidant effects of glutathione and IGF in a hyperglycaemic cell culture model of fibroblasts: Some actions of advanced glycaemic end products (AGE) and nicotine. Endocr, Metab Immune Disord - Drug Targets.

[94] Figuero E, Soory M, Cerero R, Bascones A (2006). Oxidant / antioxidant interactions of nicotine, Coenzyme Q10, Pycnogeneol and phytoestorgens in oral periosteal fibroblasts and MG63 osteoblasts. Steroids.

